# Electrospun Amphiphilic Nanofibers as Templates for In Situ Preparation of Chloramphenicol-Loaded Liposomes

**DOI:** 10.3390/pharmaceutics13111742

**Published:** 2021-10-20

**Authors:** Ivo Laidmäe, Andres Meos, Irja Alainezhad Kjærvik, Sveinung G. Ingebrigtsen, Nataša Škalko-Basnet, Kalle Kirsimäe, Tavo Romann, Urmas Joost, Vambola Kisand, Karin Kogermann

**Affiliations:** 1 Institute of Pharmacy, Faculty of Medicine, University of Tartu, Nooruse 1, 50411 Tartu, Estonia; ivo.laidmae@ut.ee (I.L.); andres.meos@ut.ee (A.M.); 2 Department of Immunology, University of Tartu, Ravila 19, 50411 Tartu, Estonia; 3 Department of Pharmacy, UiT The Arctic University of Norway, N-9037, Universitetsvegen 57, 9037 Tromsø, Norway; irjakj@gmail.com (I.A.K.); sveinung.ingebrigtsen@gmail.com (S.G.I.); natasa.skalko-basnet@uit.no (N.Š.-B.); 4 Department of Geology, University of Tartu, Ravila 14A, 50411 Tartu, Estonia; kalle.kirsimae@ut.ee; 5 Institute of Chemistry, Faculty of Science and Technology, University of Tartu, Ravila 14A, 50411 Tartu, Estonia; tavo.romann@ut.ee; 6 Institute of Physics, Faculty of Science and Technology, University of Tartu, Ravila 14C, 50411 Tartu, Estonia; urmas.joost@gmail.com (U.J.); vambola.kisand@ut.ee (V.K.)

**Keywords:** liposome, electrospinning, amphiphilic nanofibers, film hydration, drug release, chloramphenicol

## Abstract

The hydration of phospholipids, electrospun into polymeric nanofibers and used as templates for liposome formation, offers pharmaceutical advantages as it avoids the storage of liposomes as aqueous dispersions. The objective of the present study was to electrospin and characterize amphiphilic nanofibers as templates for the preparation of antibiotic-loaded liposomes and compare this method with the conventional film-hydration method followed by extrusion. The comparison was based on particle size, encapsulation efficiency and drug-release behavior. Chloramphenicol (CAM) was used at different concentrations as a model antibacterial drug. Phosphatidylcoline (PC) with polyvinylpyrrolidone (PVP), using ethanol as a solvent, was found to be successful in fabricating the amphiphilic composite drug-loaded nanofibers as well as liposomes with both methods. The characterization of the nanofiber templates revealed that fiber diameter did not affect the liposome size. According to the optical microscopy results, the immediate hydration of phospholipids deposited on the amphiphilic nanofibers occurred within a few seconds, resulting in the formation of liposomes in water dispersions. The liposomes appeared to aggregate more readily in the concentrated than in the diluted solutions. The drug encapsulation efficiency for the fiber-hydrated liposomes varied between 14.9 and 28.1% and, for film-hydrated liposomes, between 22.0 and 77.1%, depending on the CAM concentrations and additional extrusion steps. The nanofiber hydration method was faster, as less steps were required for the in-situ liposome preparation than in the film-hydration method. The liposomes obtained using nanofiber hydration were smaller and more homogeneous than the conventional liposomes, but less drug was encapsulated.

## 1. Introduction

Liposomes are spherical vesicles consisting of an aqueous core surrounded by one or several phospholipid bilayers. Liposomes have played a major role in drug delivery research and product development as part of nanomedicine. One of the biggest challenges in liposome preparation is obtaining a product which has a monodispersed size distribution and decent stability [1]. Increasingly important are the issues related to scaling-up for industrial production and scaling-down for point-of-care applications, which have motivated improvements to the conventional processes and have also led to the development of novel methods of liposome formation. For topical applications, in-situ preparation of the formulation at the site of administration is a convenient approach that enables avoiding stability issues during storage. This approach is widely used for in-situ gel systems for topical drug delivery (e.g., eye, skin) [2,3,4].

Although liposome formation may be spontaneous, some mechanical agitation is usually required. Four classical methods for manufacturing liposomes are generally known, namely, the mechanical dispersion method of thin film hydration [5], the solvent-dispersion method of solvent injection [6,7], the detergent-removal method [8] and reverse-phase evaporation [9]. The major difference between the various methods is the way lipids are dried and isolated from organic solvents and then redispersed in an aqueous medium [1,10]. Often, the filter extrusion of hydrated liposomes is applied to obtain liposomes more homogeneous in size [11]. It has been shown that various liposome preparation methods exist and these enable the obtaining of liposomes with very different properties [12,13,14].

Indeed, during recent years several new approaches have emerged. For example, heating methods [15] and microfluidic methods have been applied in the preparation of self-assembled nanosized drug delivery systems (DDSs) [1,16]. As traditional methods of preparation suffer from high batch-to-batch variation and polydispersity due to uncontrollable synthesis, in the microfluidics method, the mixing rate, heat, and mass transfer are more precise, hence synthesis, in these devices, is more controlled [17]. A novel strategy for using electrospun composite nanofibers as templates in fabricating liposomes has been introduced by Yu et al. [18]. This approach exploits the hydration of phospholipids deposited on electrospun nanofibers for the formation of liposomes. The templating and confinement properties of the nanofibers enabled the spontaneous self-assembly of phosphatidylcholine (PC), forming liposomes. Electrospinning is widely used and one of the best methods of producing non-woven polymer fibers with nano- to microscale diameters [19]. Similarly, as a solution blow-spinning method [20], the electrospinning method provides ease of operation and scalability for commercial production, and, additionally, a large variety of materials are suitable for electrospinning [19]. Electrospun fibers have a very unique structure, with several advantages (tunable porosity, small pore size, high surface-to-volume ratio, the potential to incorporate different drug molecules, excellent mechanical properties) [21] and applications in different fields of science, such as drug release and delivery [22], including RNA delivery [23], tissue engineering and wound healing, filtration, catalysis, batteries and supercapacitors [24]. One of the applications is the use of electrospun nanofibers as templates for making structures at the nano-to-micro scales. To date, only a few studies have investigated such liposomes that have been self-assembled from hydrated amphiphilic nanofibers [18,25,26,27] and to whose structures active substances have been added [25,26,27]. To the authors knowledge, none of these studies have used this approach in a comparison with the traditional film-hydration method or for the preparation of antibacterial-drug-chloramphenicol (CAM) loaded liposomes. For the first time, we have tried to understand the effect of drug-loaded electrospun fibers’ diameters and such fibers’ compositions (drug concentration; PC concentration) on the properties of drug-loaded liposomes (liposome diameter). It is relevant to understand whether such an approach can be used for the in-situ preparation of topical DDSs in order to avoid possible stability problems during storage, and/or large-scale manufacturing problems. Hence both the chemical instability of antibacterial agents (e.g., antibiotics, antimicrobial peptides), as well as the physical instability of the liposomes carrying them could be avoided with such in-situ preparation of antibiotic-loaded liposomes. Liposomes, as DDSs for antibiotics, have been widely discussed in the literature [28] and several liposome drug products for topical delivery are currently on the market, such as ketoprofen gel or amphotericin B gel (reviewed in [29]). The known major advantages of topical antibacterial liposomal DDSs are their avoidance of the systemic absorption of antibiotics and the sustainability with which they release drugs into the epidermis. The most common disadvantages of liposomal DDSs include high production costs, the instability of their dispersions, low encapsulation efficiency and the short half-life of the drug they contain [30,31,32].

Liposomes’ properties differ considerably with lipid composition, surface charge, size, and the method of their preparation [32]. The main characteristics of a liposome to study and compare are its size, transition temperature, surface charge, fluidity, lamellarity, stability and encapsulation efficiency [12,13]. These different physicochemical properties (e.g., lamellarity, surface charge, shape and size) also largely affect the behavior of the vesicles. The characterization of liposomes is important in understanding and predicting how they will act in prospective applications. In addition, chemical stability (degradation of phospholipids structures) and physical stability (uniformity of size distribution and encapsulation efficiency) are crucial in formulating liposomes for drug delivery applications, since these determine the shelf life of liposomes and, thus, the scope of their applications. Although it is known that all liposome preparation methods involve the four following steps: 1. Drying-down lipids from organic solvent. 2. Dispersing the lipids in an aqueous media. 3. Purifying the resultant liposomes. 4. Analyzing the final product [32]. It is expected that liposomes prepared by different methods may also have different properties. It is indicated that the use of liposomes might be useful for improved local activity while diminishing the percutaneous absorption of the drug. Therefore, while formulating liposome-based DDSs for topical application, it is important that the formulation is stable and provides the desired drug-release behavior [33].

The main objective of the present study was three-fold: (i) to electrospin and characterize amphiphilic nanofibers consisting of an antibiotic and a liposome-forming agent; (ii) to prepare and characterize the antibiotic-loaded liposomes by the hydration of phospholipids deposited on the electrospun amphiphilic nanofibers and by conventional film-hydration methods; (iii) and to compare the relevant properties of the prepared liposomes and understand the advantages and disadvantages of the novel nanofiber-hydration liposome-preparation method. CAM and PC were used as a model antibacterial agent and liposome-forming agent, respectively. Solid-state and morphology characterizations of the electrospun nanofiber matrices, as templates, was performed in order to understand whether the properties of templates affect the properties of the liposomes that self-form during hydration.

## 2. Materials and Methods

### 2.1. Materials

Chloramphenicol, CAM (Sigma-Aldrich, Chemie GmbH, St. Louis, MO, USA; Lots SLBH3546V and 120M0175V) was used as a model antibiotic agent. Soybean phosphatidylcholine, PC (Lipoid S-100, Lipoid GmbH, Ludwigshafen, Germany) was used as a liposome-forming agent, although PC is known to have several important applications in the human body [34] and has been used as a drug molecule [35]. Polyvinylpyrrolidone, PVP (Kollidon 90F K90, BASF SE, Germany; Lot 82296056PO) was applied as a carrier polymer for electrospinning due to its good electrospinnability in different solvents [36]. Hence it was easy to find a mutual solvent (ethanol) that dissolved the drug (CAM), liposome-forming agent (PC) and the polymer (PVP) and to obtain a homogeneous electrospinning solution. Rhodamine 123 (Sigma-Aldrich, Chemie HmbH, St. Louis, MO, USA; Lot BCBL8890V) was used as a fluorescent marker in the fluorescence microscopy studies. Solvents (ethanol, EtOH, 96.5%; methanol, HPLC grade, ≥99.9%) were of analytical grade, obtained from Sigma-Aldrich Inc. (Darmstadt, Germany) and selected for their safety and practical biomedical applications (e.g., wound healing).

### 2.2. Methods

#### 2.2.1. The Preparation of Electrospun Amphiphilic Nanofibers

Different nanofiber compositions were initially tested by varying the amounts of carrier polymer (PVP) and liposome-forming agent (PC) in the nanofibers; some of the tested compositions are listed in Table 1. In order to keep the electrospinning conditions similar, the PVP concentration was kept constant in all solutions (7.3% *w*/*v*). For example, for making NF1, 0.365 g of PVP was dissolved in 5 mL of EtOH. For making NF6, the amount of PC and PVP were 0.33 g and 0.66 g, respectively, and these solid materials were dissolved in 9 mL of EtOH. For NF7 preparation, the amount of PC and PVP were 0.6 g and 0.4 g, respectively, and these solid materials were dissolved in 5.5 mL of EtOH. CAM, in 3.75% and 18.75%, concentrations was used as a model drug in the drug-loaded nanofiber preparation. To make the drug-containing solutions for the electrospinning of drug-loaded fibers, CAM (0.0375 g or 0.1875 g) and PC (0.3 g) were dissolved in EtOH (9 mL or 7 mL) prior to adding the PVP (0.66 g or 0.5125 g, respectively). All electrospinning solutions were allowed to stir on a magnetic stirrer, at room temperature, for 24 h before use.

The polymeric nanofibers (pure PVP), as well as the empty and drug-loaded amphiphilic nanofibers, were prepared using an ESR200RD robotized electrospinning system (NanoNC, Seoul, Korea). The electrospinning process was optimized by varying the electrospinning conditions and finally carried out using a 2.5-mL syringe with a 25G blunt needle at an injection rate of 5 mL/h, provided by an automatic syringe pump. The total volume of the electrospinning solution was 2.5 mL. The voltage was varied between 10–12 kV. The air humidity and temperature during electrospinning were 18–20% and 22–25 °C, respectively. The distance between the needle and the collector plate was 11 cm. The electrospun nanofibers were collected onto an aluminum foil and put into ziploc bags. All samples were kept in a refrigerator (8 °C) and at 0% RH above silica gel in a desiccator for 12 h before further study, to reduce the effects of humidity.

#### 2.2.2. Preparation of Liposomes

**Figure 1:**
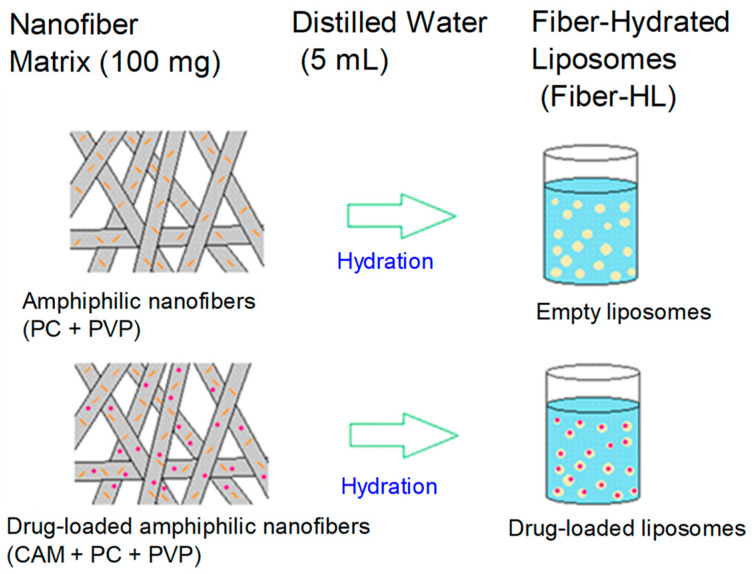
Schematic representation of the prepared liposome dispersions from hydrated nanofibers; amphiphilic nanofibers consisting of phosphatidylcholine (PC) and polyvinylpyrrolidone (PVP) were hydrated to form empty liposomes, and drug-loaded amphiphilic nanofibers consisting of chloramphenicol (CAM), PC and PVP were hydrated to form the drug-loaded amphiphilic liposomes.

Liposomes were prepared by two different methods: (i) an electrospun-nanofiber-hydration method, producing fiber-hydrated liposomes (fiber-HL), and (ii) a conventional film-hydration method, producing film-hydrated liposomes (film-HL). The first method uses electrospun composite nanofibers as templates in fabricating liposomes and was introduced by Yu et al. [18]. Hence, this method allows in-situ liposome preparation. The electrospun nanofibers were hydrated to form empty and drug-loaded liposomes (Figure 1). A total of 100 mg of nanofibers (NF4-NF7) (Table 2) were hydrated with 5 mL of distilled water and vortexed (Vortex-Genie 2, G560E, speed range 600–2700 RPM, Scientific Industries Inc., Bohemia, NY, USA) for 5 min (dial setting 5 of 10) until the nanofibers were dissolved and white and homogeneous liposome dispersion was obtained. The reproducibility of the liposome preparation was confirmed using replicates (n = 5–10).

Film hydration was performed to prepare liposomes with two different CAM concentrations (11% and 62.5% CAM/PC) (Table 2). Shortly thereafter, CAM (24.9 mg or 125 mg) and PC (200 mg) were dissolved in EtOH (20 mL) in a round-bottom flask. The EtOH was evaporated using a rotary evaporator for 20 min at 150 mbar (45 °C and 80 rpm), and subsequently for 1 h at 50 mbar (45 °C and 80 rpm). The time period was extended if needed. After the thin lipid film had dried, 10 mL of distilled water was added to the round bottom flask and its contents were manually shaken for approximately 20 min. Vortexing was used if needed. The reproducibility of the liposome-preparation method was confirmed by replication (n = 3–5). The drug–lipid ratio in the film-HL was targeted to match the ratio in the fiber-HL (Table 1). An additional size-extrusion step was also included when preparing the film-HL for better comparison with the fiber-HL, which is a known step for homogenizing samples and reducing liposome size. Syringe extrusion was performed, initially through a 0.8-µm pore-size filter (Whatman^®^ Cellulose acetate, Sigma-Aldrich, Darmstadt, Germany), and then three times through 0.45-µm pore-size filter (Whatman^®^ Cellulose acetate, Darmstadt, Germany) using an automatic syringe pump (Kd Scientific, Geneq, Inc., Holliston, MA, USA). All liposome dispersions were analyzed immediately after their preparation.

Respective physical mixtures (PMs) of the compositions were prepared, to be used as controls for the solid-state analyses. The PMs consisted of the same materials that were used in the preparation of the electrospinning solutions, without any solvent (CAM, PC or PVP). For their preparation, a mortar and pestle were used and the geometric dilution method was applied in order to be successful and obtain homogeneous mixtures.

#### 2.2.3. Electrospun Nanofibers’ Characterization

##### Surface Topography and Morphology

Electrospun nanofibers were imaged using a high-resolution scanning electron microscope, revealing both their diameters and surface morphologies (SEM, Zeiss EVO MA, Oberkochen, Germany). Samples were mounted on aluminum stubs with silver paint and magnetron sputter-coated with a 3-nm gold layer in an argon atmosphere prior to SEM microscopy.

##### Solid State Characterization and Sample Homogeneity

*X-ray diffractometry (XRD).* X-ray diffraction (XRD) patterns of all starting materials and electrospun nanofibers were obtained by using an X-ray diffractometer (D8 Advance, Bruker AXS GmbH, Karlsruhe, Germany). Pure powders (CAM, PC, PVP), PMs and electrospun matrices were measured directly from a powder holder. The XRD experiments were carried out in a symmetrical reflection mode (Bragg–Brentano geometry) with CuK_α_ radiation (1.54 Å). The angular range was from 5° 2-*theta* to 40° 2-*theta*, with steps of 0.02° 2-*theta*. The scattered intensities were measured with a 165-channel LynxEye one-dimensional detector. The operating voltage and current were 40 kV and 40 mA, respectively.

*Raman Scattering Microspectroscopy (Raman Mapping).* In order to monitor the drug and PC distribution within the fiber samples, Raman mapping was performed using a Reinshaw InVia micro-Raman spectrometer (Reinshaw, Charfield, England) with CCD camera (1040 × 256) and 785-nm diode laser excitation. An exposure time of 100 s and a 50× objective (laser spot size 5 × 20 µm) were used for the measurements. Raman mapping data were collected on a 90 × 90 μm area of the fibers in the spectral range of 672.1 to 1765.8 cm^−1^ with 0.6 cm^−1^ resolution. The maps were collected at a 1.2-μm step size in both direction and consisted of 5520 points. One spectrum acquisition took 20 s and accumulated twice in each mapping point. Additional Raman spectra from pure samples (CAM, PVP, PC), PMs and nanofiber samples were collected for the solid-state analysis using the same Raman spectrometer.

*Attenuated Total Reflection Fourier Transform Infrared Spectroscopy (ATR-FTIR).* The infrared spectra of the electrospun matrices and pure materials/PMs were collected by attenuated total reflectance Fourier transform infrared (ATR-FTIR) spectroscopy (Shimadzu IRPrestige-21, Shimadzu Corp, Kyoto, Japan). The spectroscope was equipped with a Specac Golden Gate ATR crystal composed of a diamond ATR and a ZnSe focusing element (Specac Ltd., Orpington, UK). The measurements were performed in a spectral range from 600 to 4000 cm^−1^ with 10 accumulations and a resolution of 4 cm^−1^. An IR solution software (Shimadzu, Kyoto, Japan) was used for data collection and pretreatment with baseline correction and normalization. All spectra are normalized and off-set in the *y*-axis for clarity.

#### 2.2.4. Liposome Characterization

##### Surface Topography and Morphology

The surface topographies and morphologies of different liposomes (fiber-HL and film-HL) were investigated using optical light microscopy CETI MAGTEX (Medline Sci., Chalgrove Oxon, UK) and fluorescence microscopy (Fluorescence Microscope System, DM 5500 B, Leica Microsystems, IL, USA). The concentrated liposome dispersions were prepared by self-deposition in a vacuum for 10 min and imaged using optical microscopy. Fluorescence microscopy with rhodamine 123 (Sigma-Aldrich Inc. (Darmstadt, Germany) was used to visualize the liposomes and investigate their morphologies in more detail. For fluorescence imaging, a rhodamine 123 solution (0.01 mM) in distilled water was used. Liposome dispersion (5 mL) was ultracentrifuged using a Beckman Coulter ultracentrifuge (Beckman Coulter Inc., Brea, CA, USA) with a SW55 Rotor at 50,000 rpm (for 1 h at 4 °C). The liposome pellet was resuspended in a rhodamine 123 solution (1 mL) and incubated for at least 1 h before imaging with the fluorescence microscopy.

##### Particle Size Analysis—Photon Correlation Spectroscopy (PCS)

The PCS method was used to analyze the particle sizes and particle-size distributions of the liposomes. All measurements were performed with a PCS instrument (Malvern Zetasizer Nano, Malvern Panalytical Ltd., Malvern, UK). The preparation of the samples was performed in a laminar flow cabinet to prevent contamination. To minimize the interference of nanofiber carrier polymer on the results, the liposome dispersions were ultracentrifuged immediately after the preparation and before PCS analysis and the supernatant containing polymer PVP was removed. Ultracentrifugation was performed as written in the paragraph *Surface Topography and Morphology of Liposomes*, above. All liposome dispersions were diluted 100-fold in distilled water and analyzed on the day of preparation. The particle-size analysis was performed at 22–24 °C. All liposome dispersions were analyzed in triplicate by PCS using data collection times of 10 min each. All measurements were performed in triplicate or greater.

##### Drug Encapsulation Efficiency—High-Performance Liquid Chromatography (HPLC)

To study the encapsulation efficiency of the liposomes, the fiber and film liposome dispersions (5 mL) were ultracentrifuged before the HPLC analysis, similarly to the surface morphology analysis and PCS (1 h, 50,000 rpm, 4 °C). Both the supernatant and pellet were analyzed by HPLC. The mobile phase consisted of 20 g/L phosphoric acid, methanol and water in a ratio of 5:40:55. The detector wavelength was set to 275 nm and a C18 standard column was used. The sample was diluted in methanol prior to analysis. All different measurements were performed at least in triplicate and the measurement was carried out in triplicate for each sample. To calculate the encapsulation efficiency, the following Equation (1) was used:

(1) $$Encapsulation\;efficiency\;\left(\%\right)=\frac{W_{\mathrm{pellet}}}{W_{\mathrm{total}}}\;\cdot \;100,$$

where, W_pellet_ = amount of CAM in the pellet; W_total_ = amount of CAM in the whole sample (supernatant + pellet).

##### In-Vitro Drug Release

The dialysis-tube method was used to measure the in-vitro release of the model drug, CAM, from the liposomes, together with automatic dissolution-testing equipment with paddles (Termostat-Sotax AT7, Sotax GmbH, Lörrach, Germany). The drug-release tests were performed with (i) total liposome dispersions (encapsulated and non-encapsulated free drug) and (ii) redispersed ultracentrifuged liposomes (only encapsulated drug). Additional washing steps with distilled water and redispersion in 5 mL of distilled water were performed prior to analysis. Five millilitres of both the prepared fiber-hydrated liposome dispersions (3.75% and 18.75% CAM) or of 11% film-hydrated liposome dispersion were put into a dialysis bag (molecular weight cut-off at 10 kDa, Membrane-Cel, Chicago, IL, USA). For the film-hydrated liposome dispersion consisting of 62.5% CAM, only 1 mL of liposome dispersion was used for the drug-release study. The bag was closed at both ends and placed in 500 mL of fresh PBS medium (pH 7.4) at 37 °C. Testing was performed under sink conditions and constant movement of50 rpm. Phosphate buffer (1 × PBS) with pH 7.4 (typical for blood) was used as a biorelevant buffer for wound-healing applications in order to mimic wound-bed conditions [37]. Additional weight (magnetic stirrers) was used with the dialysis bag in order to conduct the measurements. At predetermined time intervals, samples were taken and analyzed using a UV spectrophotometer at a wavelength of 275 nm. All measurements were performed at least in triplicate using 2–4 parallel measurements. The release test was continued for up to 72 h.

##### Stability Testing during Storage

The stability of the fiber-hydrated liposomes (fiber-HL1, fiber-HL2, fiber-HL3, fiber-HL4) was tested during short-storage testing at room temperature (RT) (23 ± 1.5 °C) and at fridge temperature (FT) (7.2 ± 0.7 °C). Five millilitres of liposome dispersion was prepared for each formulation and duplicate samples were stored for each environmental condition. The size and polydispersity index (PDI) values of the liposomes were recorded at specified timepoints (24 h, 48 h, 72 h and 1 month), as explained above in the paragraph *Particle-Size Analysis—Photon Correlation Spectroscopy (PCS)*.

#### 2.2.5. Data Analysis

##### Diameter Measurement of the Electrospun Nanofibers

The SEM micrographs were analyzed using the image-processing computer program ImageJ, version 1.52n [38] to calculate the mean diameter of each of the fiber compositions. The diameters of 100 randomly selected nanofibers were measured on SEM micrographs to calculate the mean diameter.

##### Drug-Release Study

Cumulative CAM release values (µg/mL) are provided. Analyses were performed in Microsoft Excel 2013.

##### Statistics

When applicable, the calculation of the arithmetic means, standard deviations (S.D), one-way ANOVAs and *t*-tests, at a confidence level of 95%, were performed using Microsoft Excel 2013 and OriginPro 8.5.0 (Originlab Corporation, Northampton, MA, USA). Two sample *t*-tests, assuming equal or unequal variances (depending on the results of the prior F-test with MS Excel 2013 software) were performed. In case of multiple comparisons, Holm’s method was used for adjusting p-values. OriginPro was also used to prepare the illustrations.

## 3. Results and Discussion

### 3.1. Characterizing Electrospun Amphiphilic Fibers as Templates for Liposome Formation

#### Morphology of Fibers

**Figure 2:**
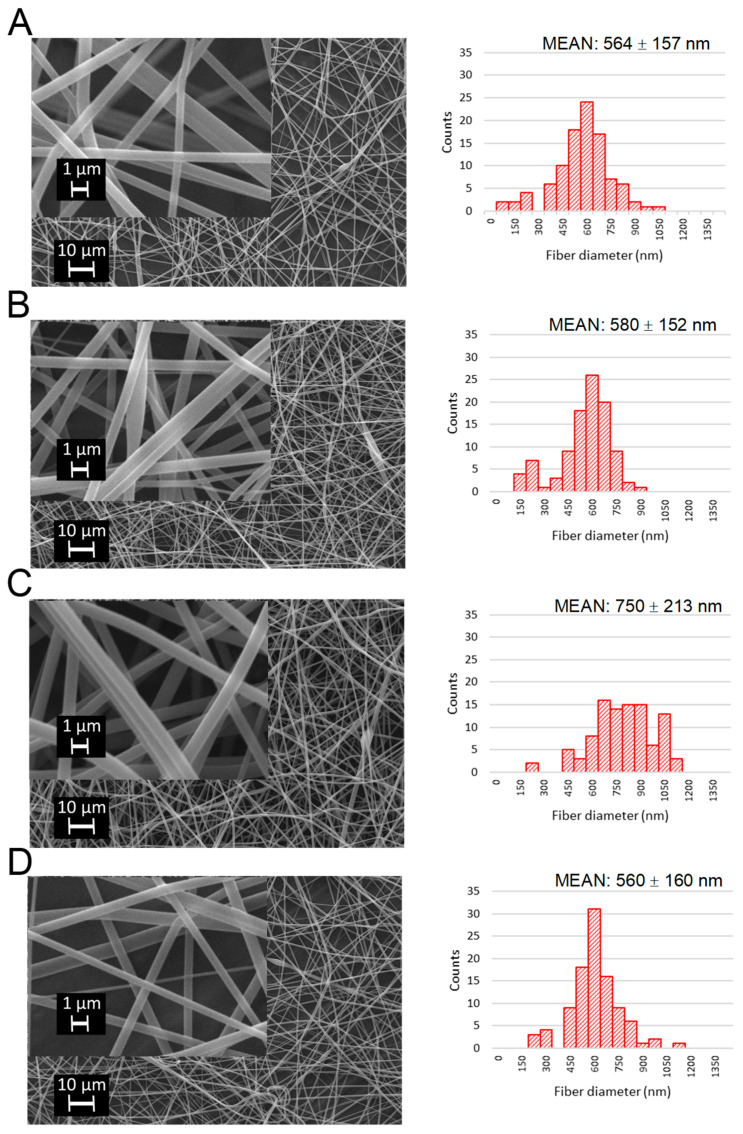
Scanning electron microscopy (SEM) micrographs of electrospun fibers with two different magnifications. (**A**) 3.75% CAM + 65.2 polyvinylpyrrolidone (PVP) and 31.1% phosphatidylcholine (PC) (NF4); (**B**) 18.75% CAM + 52.5 PVP and 30.1% PC (NF5); (**C**) 66.3% PVP and 33.8% PC (NF6); (**D**) 40.0% PVP and 60.0% PC (NF7) and their mean fiber diameters and diameter distributions as histograms. Mean fiber diameters measured n = 100. Key: NF—nanofibers.

The SEM micrographs confirmed that the processing parameters were optimal for the electrospinning of amphiphilic nanofibers both with and without CAM (Figure 2). In addition, the pure polymeric fibers and fibers with different amount of PC were successfully electrospun resulting in the formation of nanofibers (Supplementary Figure S1). The fibers had smooth and uniform surface with no beads. Electrospun fibers can be classified as larger nanofibers and for some compositions as microfibers, with mean fiber diameters (±SD) ranging from 560 ± 160 to 750 ± 213 nm. Visually, no differences were observed as regards the processability of the fibers when different formulations—pure polymeric versus PC and/or drug-containing—were electrospun (reference is made to Table 1). Mean fiber diameters and diameter size distributions were measured by SEM, since it was hypothesized that fiber diameter may significantly affect the size of the formed liposomes during the hydration step. Additionally, different compositions of the formulations may affect the diameters of the electrospun fibers. As known from the literature, it is possible to manipulate the size of the self-assembled liposomes by varying the content of PC in the nanofibers [18]. In the present study, we varied the amounts of PC and CAM, and in order to have reproducibly successful electrospinning with different formulations, PVP amounts were kept constant (Table 1). Our main interest was to understand whether the amount of drug and PC affects the formation of liposomes from electrospun fiber mats, as well as whether the composition of the fibers and their diameters influence the sizes of the formed liposomes.

In our study, the fiber diameter size distributions of drug-loaded and unloaded fiber mats mainly followed the normal size distribution. The largest fiber diameter and standard deviation was observed for NF6 (750 ± 213 nm) (Figure 2) which was statistically significantly different from other formulations. Interestingly, all other fiber diameters were almost the same size. Indeed, there were large variations in the fiber diameters of the different formulations which may be the result of different compositions, but also different environmental conditions during electrospinning. Hence, we were not able to make any clear correlations between the added CAM amount and electrospun fiber diameter. Song et al. have shown a trend wherein greater amounts of Fe_3_O_4_ nanoparticle, when incorporated into the electrospun fibers, leads to larger fiber diameters. However, also huge variations appeared in their study in their fibers’ diameters and no linear correlation can be seen [26]. Furthermore, PC concentration also did not show a clear correlation with the diameter. Yu et al. have concluded that the fiber diameter increases with the PC concentration, but the data presented in their publication show that the addition of PC, initially, on the contrary, decreased fiber diameter and, only at higher concentrations, increased fiber diameter [18].

### 3.2. Solid State Characterization of Fibers

#### 3.2.1. XRD and Raman Mapping

**Figure 3:**
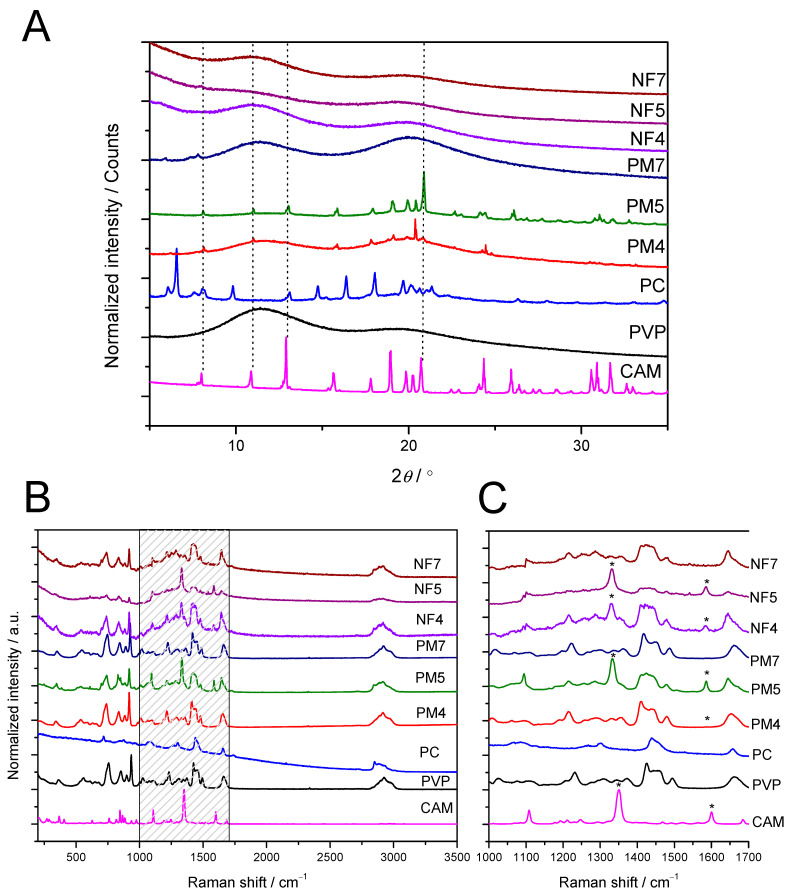
Solid-state analysis of electrospun nanofiber templates. (**A**) Normalized X-ray diffraction (XRD) patterns and (**B**) the Raman spectra of pure CAM, PVP and PC and the PMs and electrospun nanofibers of CAM, PVP and PC in the different formulations NF 4, NF 5 and NF 7 (reference is also made to Table 1). (**C**) Figure enlargement of the graded area of Figure 3B, showing the Raman spectra of pure CAM, PVP and PC, PMs and the electrospun nanofibers of CAM, PVP and PC in the different formulations NF 4, NF 5 and NF 7 in the spectral region from 1000 to 1700 cm^−1^. The dotted lines on XRD diffractograms and asterisks (*) on the Raman spectra point out the characteristic XRD reflections and Raman peaks of CAM, respectively. Key: CAM—chloramphenicol; NF 4—nanofibers consisting 3.75% CAM + 66.25% polyvinylpyrrolidone (PVP) and 30% phosphatidylcholine (PC); NF 5—nanofibers consisting 18.75% CAM + 51.25% PVP and 30% PC; NF7—nanofibers consisting 40.00% PVP and 60.00% PC; PC—phosphatidylcholine; PM4-physical mixture of 3.75% CAM + 66.25% polyvinylpyrrolidone (PVP) and 30% phosphatidylcholine (PC); PM5—physical mixture of 18.75% CAM + 51.25% PVP and 30% PC; PM7—physical mixture of 40.00% PVP and 60.00% PC; PV—polyvinylpyrrolidone.

Drug-loaded fibers are known to consist active pharmaceutical ingredients in an amorphous form [39,40,41]. It is due to the fact that the solvent evaporation is fast enough to avoid any recrystallization of drug during the electrospinning process. Also, the results from the present study revealed that, during electrospinning, CAM transformed into an amorphous form. No characteristic crystalline reflections were observed on the XRD diffractograms (Figure 3A). Despite the amount of CAM, all drug-loaded fibers (3.75% vs 18.75%) showed the same behavior. The corresponding PMs verified that the crystalline CAM amount was easily detected from the mixtures and confirmed the detection limit for XRD. Furthermore, also Raman mapping results supported the XRD findings (Figure 3B). The characteristic Raman peaks of CAM are shown in Figure 3C and highlighted with stars. Similar to our XRD analysis, CAM was detected in a Raman spectrum in its crystalline form in PMs. Peak shifts, as well as decreases in intensity, were observed, confirming the presence of amorphous CAM within electrospun fiber mats. According to the literature, CAM has characteristic stretches at 1350 and 1601 cm^−1^ in its Raman spectrum, which are assigned to N–O_2_ symmetric stretching and ring stretching, respectively [42]. PVP has characteristic Raman peaks at 1427 and 1658 cm^−1^, assigned for CH2 scissor and amide, respectively [43,44]. None of the excipients showed spectral interference in these regions. PVP did not show much change during electrospinning, as its characteristic Raman peaks were unchanged. PVP is a semi-crystalline polymer. The amount of PC within the fibers and PMs was too low and not detected in XRD or Raman spectroscopy, whilst its solid-state changes could not be monitored with these techniques. As a verification, amorphous CAM was also prepared by quench-cooling the melt in liquid nitrogen and its Raman spectra collected, as reported previously [39]. The spectral features between the Raman spectra of drug-loaded NFs and amorphous CAM matched, confirming the presence of amorphous CAM within the NFs.

**Figure 4:**
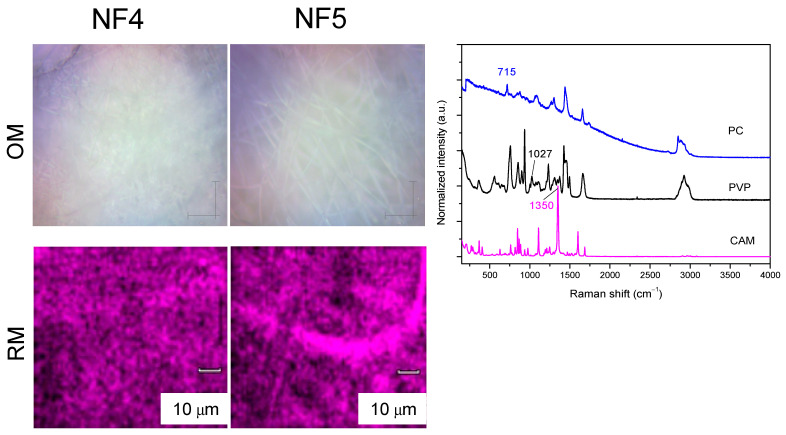
Distribution of chloramphenicol (CAM) in the electrospun nanofiber mats, measured by Raman scattering microspectroscopy (Raman mapping) and, for comparison, optical microscopy images of the fibers are provided. The characteristic Raman peaks for CAM (1350 cm^−1^), PVP (1027 cm^−1^) and PC (715 cm^−1^) are emphasized on the figure. Key: CAM—chloramphenicol; NF 4—nanofibers consisting 3.75% CAM + 66.25% polyvinylpyrrolidone (PVP) and 30% phosphatidylcholine (PC); NF 5—nanofibers consisting 18.75% CAM + 51.25% PVP and 30% PC; OM—optical microscopy images; PC—phosphatidylcholine; PVP—polyvinylpyrrolidone; RM—Raman mapping images.

As a next step, it was of interest to confirm the drug distribution within the fiber mat and within the fibers. For this purpose, Raman mapping was used. The characteristic peaks of CAM, PVP and PC were selected and mapping was performed (Figure 4). It was seen that fibrous structure was obtained using a characteristic CAM peak. This finding is supported by the observation that fibers were formed from a homogeneous solution and the electrospinning conditions thereof were optimized; therefore, the drug was evenly distributed within the fibers. Due to collection onto the collector plate, there were, of course, variations in drug concentration depending of the site of collector plate; but, since the drug concentration was estimated in a solid state, the exact drug amount matched nicely with the theoretical CAM amount (confirmed by HPLC). 

#### 3.2.2. ATR-FTIR Spectroscopy

**Figure 5:**
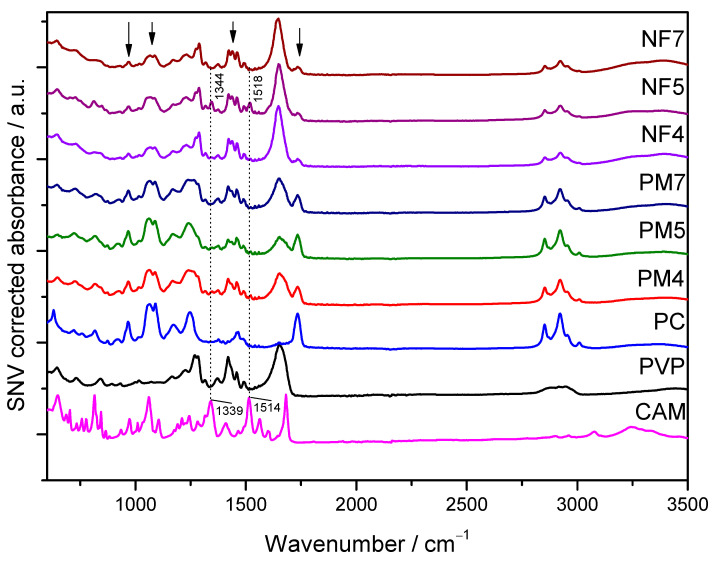
Attenuated total reflection (ATR) Fourier-transform infrared spectroscopy (FT-IR) spectra of pure chloramphenicol (CAM), polyvinylpyrrolidone (PVP), phosphatidylcholine (PC), electrospun fibers (NF) and their respective physical mixtures (PMs). Reference is also made to Table 1. The dotted line points out the characteristic IR peaks of CAM and the arrows point out the characteristic IR peaks of PC.

Due to our more complex system, consisting of three components (CAM, PVP and PC), their IR spectra were also collected. FTIR analyses were conducted to confirm the presence of different components and understand more about their physicochemical interactions within electrospun fibers. Hydrogen bonding interactions can be revealed also in Raman spectra, but since IR and Raman are complementary techniques, more deep understanding can be obtained when these techniques are used in parallel. Characterization using ATR-FTIR spectroscopy revealed that unlike previous methods this method allowed to distinguish all three components using their IR spectra: CAM, PC and PVP (Figure 5).

In the spectra of electrospun fibers, primarily PVP was detected, whilst, in PMs, more pronounced PC peaks were observed (Figure 5). It is difficult to obtain a homogeneous physical mixture (PM) between the PC and solid materials, and this was also seen in the spectra of the PMs, which showed the largest variations. The expected characteristic IR peaks of crystalline CAM, which were easily distinguished from excipients, appeared at 1514 cm^−1^ and 1339 cm^−1^, assigned to asymmetric NO_2_ stretching (ν_as_(NO_2_)) and the symmetric stretching of NO_2_, respectively [45]. Compared to the crystalline CAM spectrum, there was a slight shift towards higher wavenumbers, which confirmed the presence of amorphous CAM in the electrospun fibers (NF5), supporting the XRD and Raman spectroscopy findings (Figure 5). This solid-state transformation and the occurrence of amorphous CAM in fibers has also been shown previously for electrospun CAM fibers with polycaprolactone (PCL) [39]. These characteristic CAM peaks had much lower intensity in the NF4 spectrum compared to NF5 spectrum. The latter was due to much lower CAM concentrations in the NF4 samples as compared with the NF5 samples.

### 3.3. Characterization of Liposomes

#### 3.3.1. Morphology and Size of Fiber-Hydrated (Fiber-HL) and Film-Hydrated Liposomes (Film-HL)

The immediate hydration of phospholipids deposited on the amphiphilic nanofibers occurred within few seconds resulting in the formation of liposome dispersions. The obtained liposomes were spherical, and optical microscopy revealed a multi-lamellar liposome structure (Supplementary Figure S2). Similarly and as expected, the thin-film-hydration technique (introduced by Bangham et al. [5]) provided multi-lamellar liposomes. Hence, both of these methods are suitable for hydrophobic antibiotic compounds, as reported by Gomez et al. [46].

**Figure 6:**
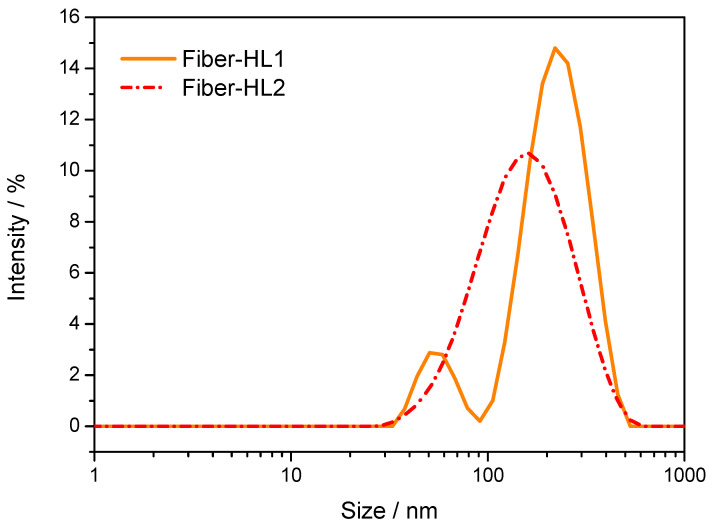
Representative particle size distribution by intensity profiles of fiber-HL1 (3.75% CAM) and fiber-HL2 (18.75% CAM). Key: fiber-HL—fiber-hydrated liposomes.

The particle size distribution of the liposomes varied from sample to sample (Table 3) but showed similar Z-average values and polydispersity profiles. There were statistically significant differences when the Z-average liposome size and PDI values were compared between different formulations (*p* ≤ 0.05). The particle size distribution of fiber-HL1 (3.75% CAM) was bimodal, with two peaks (approximately 60 and 300 nm) (Figure 6).

The particle size distribution of fiber-HL2 (18.75% CAM) appeared wide and unimodal, with a Z-average vesicle size of 132.3 ± 1.1 nm (Figure 6 and Table 3). It was seen that the presence of CAM and its concentrations within the electrospun fibers changed the properties of the formed liposomes (e.g., liposome size, stability). Despite the similarity in fiber diameters between the different formulations, the fiber mats consisting of the largest amounts of PC (NF7) resulted in the largest liposomes (fiber-HL4; liposome mean diameter above 1000 nm), as also previously reported [18] (Table 3). Interestingly, there was no clear correlation detected between the electrospun fibers’ diameters and the formed liposomes’ diameters, but the composition of the fibers (amount of CAM, PVP and PC) significantly affected the size of the liposomes. The drug-loaded fibers of higher CAM concentration resulted in smaller liposomes compared with those of lower CAM concentration. It is likely that the presence of PVP, as well as the drug (and its specific properties), changes the formation of liposomes. It is believed that the major component affecting the liposome diameter was the concentration of PC, as less PC was incorporated into the electrospun fibers with higher CAM concentrations, resulting in smaller liposomes. A prior study by Yu et al. has shown that lower PC concentration within electrospun fibers results in smaller liposomes [18]. It was confirmed that although electrospinning may result in different fiber diameters (which are affected not only by material and processing conditions, but also by environmental conditions), the formed CAM-loaded liposomes obtained, using the fiber-hydration method, were homogeneous and had a reproducible size.

The particle size analysis also showed that the vesicle size of the film-hydrated liposome dispersions (film-HL1, HL2) was very large or out of the instrument analytical range (>800 nm), therefore syringe extrusion step was included and only size reduced film-hydrated liposomes were analyzed further.

PDI values revealed that fiber-hydrated liposomes were more stable and their particle sizes more monodisperse, as the PDI values were lower compared with the film-hydrated (film-HL) and size-extruded liposomes (film-HL ex) (Table 3).

Similarly, smaller PDI values were observed with film-hydrated and sized-extruded liposomes of higher CAM concentrations. Liposomes with higher CAM concentrations were hence more stable (lower PDI values), but the mean size of the liposomes was larger (Table 3). Particle size distributions were also similar, film-HL1 had bimodal, whereas film-HL2 unimodal size-distribution profiles (Supplementary Figure S3). However, as seen in Table 3 and Supplementary Figure S3, the mean size of the film-hydrated liposomes (film-HL) was larger compared with the fiber-hydrated liposomes (fiber-HL).

**Figure 7:**
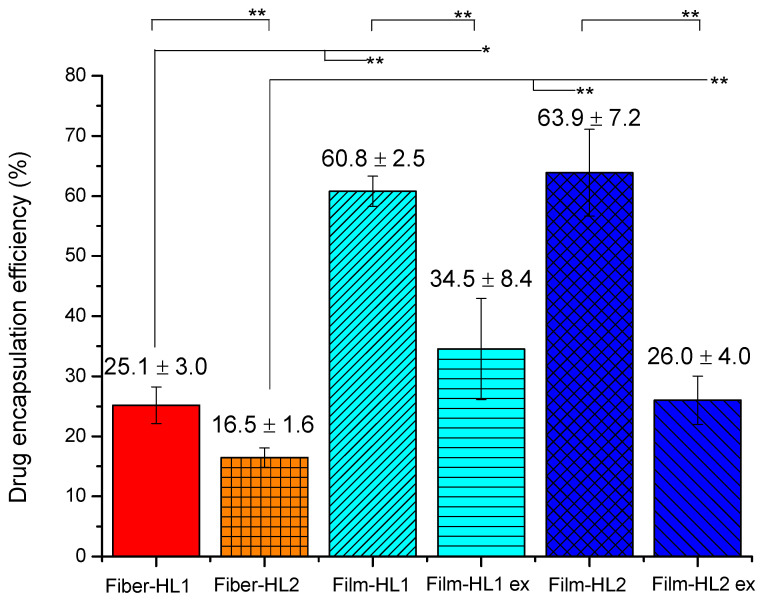
Drug encapsulation efficiency of the liposomes. The values denote the mean of drug encapsulation efficiency (%) ± SD (n = 2–3). Statistically significant differences are shown with an asterisk * (*p* < 0.05) and ** (*p* < 0.01). Key: fiber-HL—fiber-hydrated liposomes; film-HL—film-hydrated liposomes; ex—size reduced via syringe extrusion; SD—standard deviation.

Hydrophobic drugs are known to be incorporated into the lipid bilayer during liposome formation [32,47] and this has been used to design more lipophilic drug molecules for successful liposome formulations [48]. CAM is a hydrophobic antibiotic agent, hence method of liposome preparation largely affects the encapsulation of CAM [46,49] and there is a limit to how much CAM can be incorporated into the lipid membrane. The drug-encapsulation efficiency was significantly smaller for the fiber-HL compared to the film-HL (Figure 7). The size reduction of film-HL, however, made these liposomes more similar to fiber-HL and also reduced their encapsulation efficiency values. It has been shown previously that the extrusion and/or sterilization steps may lower encapsulation efficiency significantly [50]. Although it has been reported previously that the encapsulation efficiency of CAM liposomes was approximately 50% when the dual asymmetric centrifugation (DAC) method was used for homogenization and liposome preparation [49]. It is important to highlight that fiber-HL consisted of PVP in solution and during liposome formation, whereas film-HL did not. It is likely that PVP may act as a solubilizer [51] for hydrophobic CAM and increase the solubility of CAM in the solution, thus hindering its incorporation into the liposome bilayer. Moreover, Chen et al. have shown that both the ratio of PC to drug (e.g., carvedilol) and the molecular weight of PVP significantly affect a drug’s encapsulation efficiency of liposomes [27]. The higher molecular weight of PVP has been shown to decrease the encapsulation efficiency of a drug. Independent of the liposome-preparation method, less CAM was incorporated into the liposomes with a higher CAM concentration (18.75% CAM). This is, most likely, due to the solubility limits of CAM in lipids. The smaller particle size of fiber-hydrated liposomes (fiber-HL) compared to film-hydrated (film-hydrate HL) liposomes correlates with the lower encapsulation efficiency values of fiber-HL compared with film-HL, respectively. Therefore, it is likely that greater amounts of CAM in a solution will not be encapsulated into the lipid bilayer of the liposomes.

#### 3.3.2. Drug Release from Fiber-Hydrated and Film-Hydrated Liposomes

**Figure 8:**
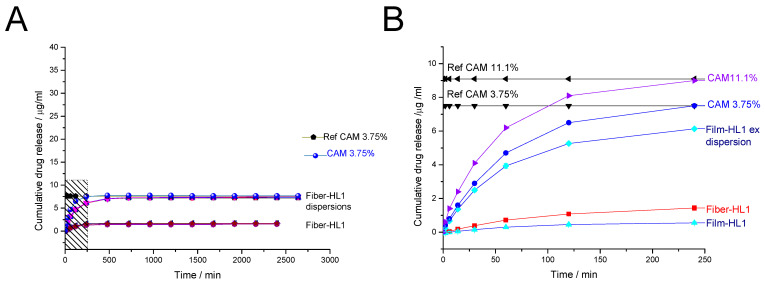
In-vitro release of CAM from CAM-loaded liposome dispersions and CAM-loaded liposomes in phosphate-buffered saline (pH 7.4, n = 3–6). Cumulative CAM release (µg/mL) from (**A**) fiber-hydrated liposome dispersions with lower CAM concentrations (fiber-HL1) and fiber-HL1 samples, up to the 3000 min timepoint; (**B**) ultracentrifuged and redispersed fiber-HL1 and film-HL1 samples, up to the 250 min timepoint. As references, solutions with 3.75%, 11.1% CAM concentrations were used directly and inserted into the membrane, and their behavior was monitored over time. Key: CAM solution 3.75%—CAM solution (7.5 µg/mL) inserted into the membrane and in a dissolution bath at 37 °C; CAM solution 11.1%—CAM solution (9.1 µg/mL) inserted into the membrane and in a dissolution bath at 37 °C; fiber-HL—ultracentrifuged fiber-hydrated liposomes (resuspended in water and inserted into a membrane); film-HL—ultracentrifuged film-hydrated liposomes (resuspended in water and inserted into a membrane); film-HL1ex—filter extruded film-hydrated liposome dispersion with lower CAM concentration inserted into a membrane; Ref fiber-HL1—CAM solution with a theoretical CAM concentration of 3.75% kept in a dissolution bath at 37 °C; Ref film-HL1—CAM solution with a theoretical CAM concentration of 11.1% kept in a dissolution bath at 37 °C. Graded area shows the region that is enlarged in figure (**B**).

The release of CAM from encapsulated liposomes prepared by different methods and that of the free drug was investigated using the dialysis method. The membrane composition of the liposome and the choice of drug are known factors to influence drug release from liposomes [52], but these were kept the same between both preparation methods. Only PVP was present in the fiber-hydrated liposome dispersions but not in the film-hydrated liposome dispersions. PVP was needed for the electrospinning of CAM- and PC-loaded nanofibers and, therefore, it was present in the solution formed during the hydration of the fiber matrices and liposome self-forming. The release experiments were all performed under sink conditions. The in-vitro cumulative release profiles for the CAM-loaded liposome formulations in phosphate-buffered saline (pH 7.4) are shown in Figure 8. As expected, the drug release of free CAM was greater than that of the CAM-loaded liposomes for every formulation.

All the different formulations are not directly comparable due to their different preparation methods, but the release profiles can be compared by considering the actual drug concentrations incorporated into the systems.

Fiber-HL1 consisted 3.75% of CAM, confirmed by HPLC. It can be seen from the results that all liposome dispersions released this amount of CAM (mean CAM release of 7.3 µg/mL), but, from the pure, ultracentrifuged liposomes (fiber-HL1), approximately 4.5× less CAM was released. It is expected that CAM will be both in the supernatant as well as within the liposomes; and, initially, while monitoring the drug release from liposome dispersions, the free drug from the dispersion is diffused through the membrane. The released amount of CAM from liposomes is consistent with the HPLC finding that the fiber-HL1 liposomes had an encapsulation efficiency of 25.1% (Figure 7) and that only this amount—approximately 1.6 µg/mL—was released, (Figure 8A). Investigation of the fiber-HL2 dispersion revealed similar behavior, it consisted of 18.5% CAM and the encapsulation efficiency of liposomes was only 16.5%, according to HPLC. The liposome dispersions released their total CAM amounts (36 µg/mL), but the fiber-HL2 liposomes separated using ultracentrifugation released only 4.6 µg/mL of CAM, which is 7.8× less and in-line with the HPLC results (Supplementary Figure S4A).

In the present study, it was of interest to understand whether fiber-hydrated liposomes show similar or dissimilar behavior to film-hydrated liposomes. Hence, film-hydrated liposomes were prepared. The average CAM amount released from film-HL1 liposome dispersions was 21.1 µg/mL (the theoretical concentration of CAM was 11.1%) (data not shown), the entrapment efficiency of these liposomes was higher compared with fiber-HL1, 60.8% (Figure 7). Since the particle size distribution of film-hydrated liposomes was wide and these liposomes had heterogeneous particle size, size-extruded liposomes were prepared and compared with the fiber-hydrated liposomes. When the size extrusion step was added, the amount of CAM released was decreased from both liposome dispersions as well as from the liposomes themselves. The total amount of CAM released from the size-extruded liposome dispersions (film-HL1 ex) was 5.9 µg/mL, whilst the CAM released from ultracentrifuged and redispersed film-HL1 liposomes was 0.7 µg/mL, which was similar to or even lower than in fiber-HL1 (1.6 µg/mL) (Figure 8B). The film-HL2 liposome dispersions acted similarly; the theoretical concentration of CAM was 18.5%, hence, the CAM amount released from the size-extruded liposome dispersions was only 9.1 µg/mL, compared to 1.25 µg/mL for film-hydrated liposomes separated by ultracentrifugation and dispersed in distilled water (Supplementary Figure S4B).

#### 3.3.3. Storage Stability of Self-Formed Fiber-Hydrated Liposomes

**Figure 9:**
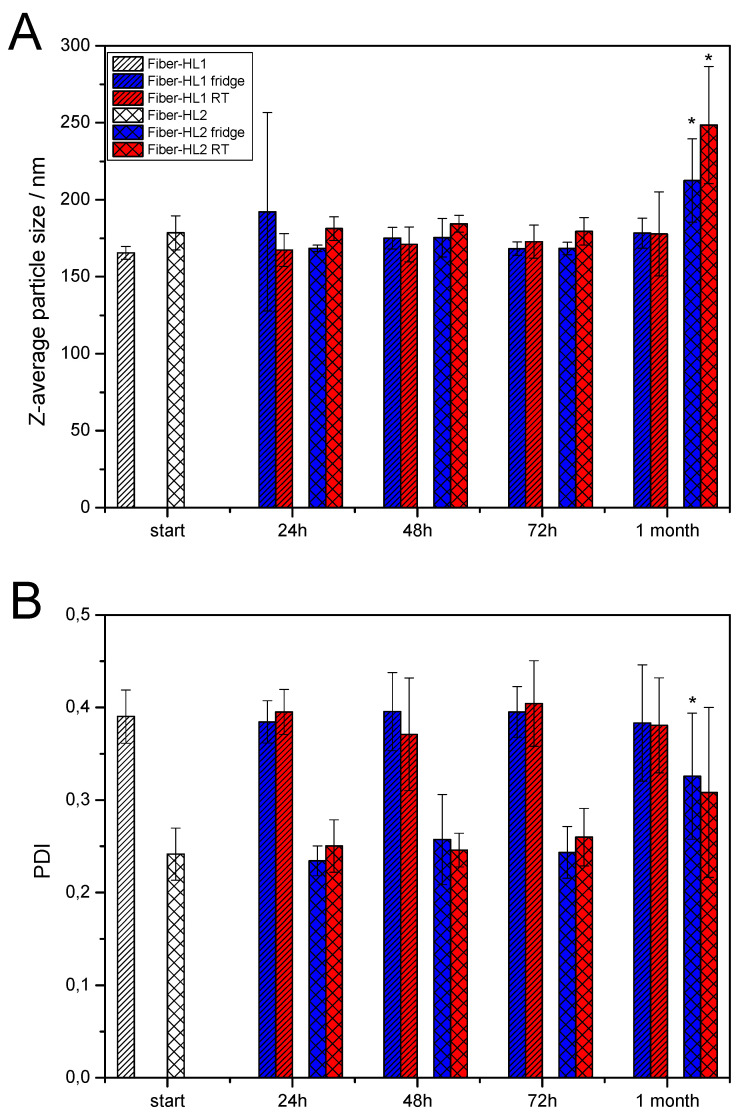
Mean size (**A**) and PDI (**B**) of self-formed fiber-hydrated liposomes (fiber-HL1 and fiber-HL2), at different timepoints, when stored at room and fridge temperatures (n = 3). Error bars show the standard deviation (SD). Statistically significant differences are shown with asterisk * (*p* ≤ 0.01). Key: Fiber-HL—fiber hydrated liposomes, fridge—samples stored in fridge; PDI—polydispersity index; RT—samples stored at room temperature.

It is known that the chemical instability of antibiotic agents needs to be assured for antibiotic drug products and most antibiotics are more stable as dry powders [53]. The advantage of using the in-situ liposome preparation method is that the antibiotic-loaded fiber matrices can be kept dry until use. However, the physical stability of such fiber-hydrated liposome dispersions was investigated in order to understand their behavior during storage. The stability tests were conducted with fiber-hydrated liposome dispersions at room and fridge temperatures for 1 month. Liposome size and PDI values were evaluated in order to understand the stability of the liposome dispersions. It was observed that the liposomes with less CAM (fiber-HL1) were more stable than the liposomes with more CAM (fiber-HL2), as the sizes of the liposomes were unchanged after 1 month of storage (Figure 9A). Fiber-HL2 liposome size increased after 1 month of storage. This result was quite expected; as the drug is released from the liposomes, this changes the properties of liposomes over time. Both the fridge- and room-temperature samples showed changes in particle size after 1 month of storage, although the size of the liposomes stored at fridge temperature were somewhat smaller compared to the liposomes stored at room temperature. Similarly, the PDI values supported the findings from particle size (Figure 9B). No statistically significant changes were observed with the fiber-HL1 liposome dispersions, but the PDI values of the fiber-HL2 liposome dispersions increased (Figure 9A,B).

The drug-free liposomes appeared to be more stable, as neither significant changes in particle size nor in PDI values were observed during storage (Supplementary Figure S5). Interestingly the storage temperature did not have any significant effect on the liposome size nor PDI value (Figure 9), although it has been shown that lower temperatures, together with cryoprotectants, indeed protect and stabilize liposomes [54].

## 4. General Discussion and Conclusions

The physiochemical analysis data here-generated allowed comparisons of drug-loaded liposomes prepared by the conventional film-hydration method with drug-loaded liposomes prepared by the fiber-hydrated method. The liposomes prepared thereby were compared for their entrapment efficiencies, vesicle sizes and polydispersities. In both methods, the liposomes self-assembled during the hydration phase, but the results confirmed that their liposome-formation mechanisms differ. It is likely that the presence of hydrophilic PVP affected the formation of the fiber-hydrated liposomes and lowered the encapsulation of the hydrophobic drug, CAM, into the liposomes. Therefore, the mean diameters of the liposomes differed considerably, as did their entrapment efficiencies. Fiber-hydrated liposomes (fiber-HL) had smaller particle sizes compared with film-hydrated and extruded liposomes. Both microscopy and PCS showed that the formed liposomes’ sizes were statistically different from each other (*p* < 0.01). It is possible that, when more extrusion steps are added after the thin film-hydration method, liposome size will be reduced even more. However it would also lessen the encapsulation of the drug, as also seen from our results and reported previously [50]. As one of its advantages, the fiber-hydration method does not require an additional extrusion step to reduce the particle size. Furthermore, this method can be used in situ and no long-term storage of aqueous dispersions is needed, which increases the stability of antibiotic-loaded liposomes.

Regardless of the preparation method used, both methods allowed the preparation of multilamellar CAM-loaded liposomes. Interestingly, both the fiber-hydrated liposomes (fiber-HL) and film-hydrated (film-HL) liposomes exhibited a polydispersity at lower CAM concentrations, and reduced heterogenicity with at higher CAM concentrations.

In conclusion, it is possible to prepare the antibiotic-loaded liposomes in situ using the hydration of phospholipids deposited on electrospun amphiphilic fibers. The advantage of the method is that the antibiotic drug is kept in dry conditions until use, which increases its chemical stability. Furthermore, the physical stability of the liposomes is assured, as administration can take place immediately after preparation.

A disadvantage of the method is that the drug-encapsulation efficiency is rather low and requires different excipients to facilitate greater drug- loads within the liposomes. Further optimization of the preparation method is required. We have proposed that the method can be used as an alternative liposome production method when the fast production of liposomes of relatively homogeneous size is needed. Another drawback of the method, lower encapsulation efficiency, was observed in the present study and may well be linked to drug molecule used and its physicochemical properties, as it is likely that the use of different drugs and/or template materials (e.g., polymer, polymer molecular weight) may lead to different encapsulation efficiencies of drugs [27].

The present nanotechnological, in-situ self-assembly approach opens up new opportunities for the fabrication, stabilization and delivery of drug-loaded liposomes.

## Figures and Tables

**Table 1:** Theoretical formulation compositions of experimental electrospun nanofibers.

Nanofiber (NF)	CAM (*w*/*w*% of the Fibers)	PC (*w*/*w*% of the Fibers)	PVP (*w*/*w*% of the Fibers)
NF 1	-	-	100
NF 2	-	20	80
NF 3	-	33.3	66.7
NF 4	3.75	30.1	66.15
NF 5	18.75	30.0	51.25
NF 6	-	33.8	66.2
NF 7	-	60	40

Key: CAM—chloramphenicol; NF—nanofiber; PC—phosphatidylcholine; PVP—polyvinylpyrrolidone.

**Table 2:** Compositions of liposome dispersions with and without chloramphenicol (CAM).

Liposome Dispersion	NF Matrix (100 mg)	H_2_O (mL)		CAM/PC Ratio
fiber-HL1	NF4	5		0.125
fiber-HL2	NF5	5		0.625
fiber-HL3	NF6	5		NA
fiber-HL4	NF7	5		NA
	**CAM (mg)**	**PC (mg)**	**H_2_O (mL)**	**CAM/PC Ratio**
film-HL1	24.9	200	10	0.125
film-HL2	125	200	10	0.625

Key: CAM—chloramphenicol; fiber-HL—nanofiber hydrated liposome; film-HL—film hydrated liposome; NA—not-applicable; NF—nanofiber; PC—phosphatidylcholine.

**Table 3:** Particle sizes of empty liposomes and liposomes loaded with chloramphenicol (CAM) and prepared using the fiber-hydration and film-hydration methods.

Liposome Dispersion	Mean Size ± SD (nm) n = 1 Experiment	Mean PDI ± SD n = 1 Experiment	Mean Size ± SD (nm) Average SD (nm) n = 3–5, Different Experiments	Mean PDI ± SD Average SD n = 3–5, Different Experiments
fiber-HL1	175.8 ± 0.5	0.38 ± 0.01	178.1 ± 3.4	0.41 ± 0.04
fiber-HL2	131.1 ± 1.2	0.21 ± 0.01	132.3 ± 1.1	0.20 ± 0.03
fiber-HL3	161.1 ± 2.6	0.38 ± 0.02	178.0 ± 25.9	0.38 ± 0.05
fiber-HL4	1189.7 ± 113.2	0.50 ± 0.13	1097.6 ± 131.9	0.39 ± 0.25
film-HL1	ND	ND	ND	ND
film-HL2	854.0 ± 25.2	0.48 ± 0.03	ND	ND
film-HL1 ex	449.9 ± 13.6	0.46 ± 0.04	434.5 ± 17.7	0.50 ± 0.13
film-HL2 ex	570.7 ± 29.2	0.41 ± 0.01	557.2 ± 48.7	0.35 ± 0.15

Key: ex—size reduced via syringe extrusion; fiber-HL—fiber-hydrated liposomes; film-HL—film-hydrated liposomes; ND—not determined; SD—standard deviation.

## Supplementary Materials

The following are available online at https://www.mdpi.com/article/10.3390/pharmaceutics13111742/s1, Figure S1: Scanning electron microscopy (SEM) images of electrospun nanofibers. (A) 100% polyvinylpyrrolidone (PVP) (NF1); (B) 20% phosphatidylcholine (PC) and 80% PVP (NF2); (C) 33.3% PC and 66.7% PVP (NF3); (D) 4% chloramphenicol (CAM), 32% PC and 64% PVP; Figure S2: Example of optical microscopy picture of prepared fiber-hydrated liposomes (fiber-HL1); Figure S3: Particle size distribution of film-hydrated liposomes (film-HL) with different chloramphenicol concentrations (CAM). Key: Film-HL1—film-hydrated liposomes with a lower amount of CAM (11.1%)**;** Film-HL2—film-hydrated liposomes with a higher amount of CAM (62.5%); ex—syringe-extruded liposomes; Figure S4: In-vitro release of CAM from CAM-loaded liposome dispersions and CAM-loaded liposomes in phosphate-buffered saline (pH 7.4, n = 3–6). Cumulative CAM release (µg/mL) from (A) fiber-hydrated liposome dispersions with a higher CAM concentration (fiber-HL2) and fiber-HL2 samples from up to the 3000-min timepoint; (B) ultracentrifuged and redispersed fiber-HL2 and film-HL2 samples from up to the 250-min timepoint. As references, solutions with 18.75% and 62.5% CAM concentrations were used directly and inserted into the membrane, and their behavior was monitored over time. For clearance purposes the reference CAM solution profiles are not shown in figure B. Key: CAM solution 18.75%—CAM solution (36.2 µg/mL) inserted into the membrane and in a dissolution bath at 37 °C; fiber-HL—ultracentrifuged fiber-hydrated liposomes (resuspended in water and inserted into a membrane); film-HL—ultracentrifuged film-hydrated liposomes (resuspended in water and inserted into a membrane); film-HL2ex—filter-extruded film-hydrated liposome dispersion with a higher CAM concentration inserted into a membrane; Ref fiber-HL2—CAM solution with a theoretical CAM concentration of 18.75%, kept in a dissolution bath at 37 °C. Graded area shows the region that is enlarged in figure B. Figure S5: Mean size (A) and PDI (B) of self-formed fiber-hydrated liposomes (fiber-HL3 and fiber-HL4) at different timepoints when stored at room and fridge temperatures (n = 3). Error bars show the standard deviation (SD). Statistically significant differences are shown with an asterisk * (*p* ≤ 0.01). Key: Fiber-HL—fiber-hydrated liposomes, fridge—samples stored in fridge; PDI—polydispersity index; RT—samples stored at room temperature.
