# Supplementary material for: Electrospun Amphiphilic Nanofibers as Templates for In Situ Preparation of Chloramphenicol-Loaded Liposomes
Source: Pharmaceutics. 2021 Oct 20;13(11):1742. doi: 10.3390/pharmaceutics13111742 (PMC8624320; doi:10.3390/pharmaceutics13111742)
Supplement: Supplementary file 1 [file pharmaceutics-13-01742-s001.zip › pharmaceutics-1406198-supplementary.pdf]

pharmaceutics

Supplementary Materials: Electrospun Amphiphilic Nanofibers as Templates for In Situ Preparation of Chloramphenicol-Loaded Liposomes

Ivo Laidmäe , Andres Meos , Irja Alainezhad Kjærvik , Sveinung G. Ingebrigtsen , Nataša Škalko-Basnet , Kalle Kirsimäe , Tavo Romann , Urmas Joost , Vambola Kisand and Karin Kogermann

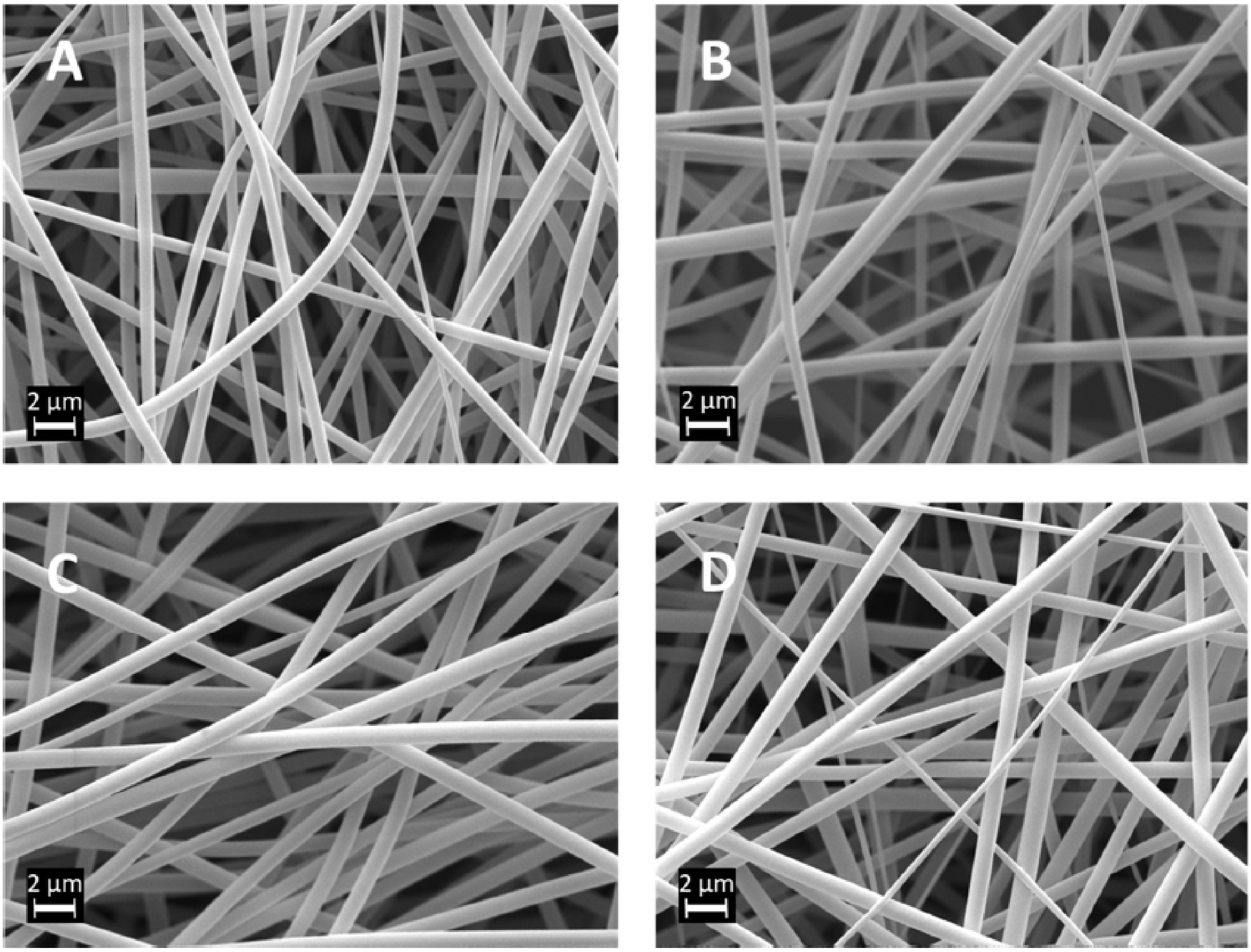

Figure S1. Scanning electron microscopy (SEM) images of electrospun nanofibers. (A) 100 % polyvinylpyrrolidone.(PVP) (NF1); (B) 20 % phosphatidylcholine (PC) and 80 % PVP (NF2); (C) 33.3 % PC and 66.7 % PVP (NF3); (D) 4 % chloramphenicol (CAM), 32 % PC and 64 % PVP.

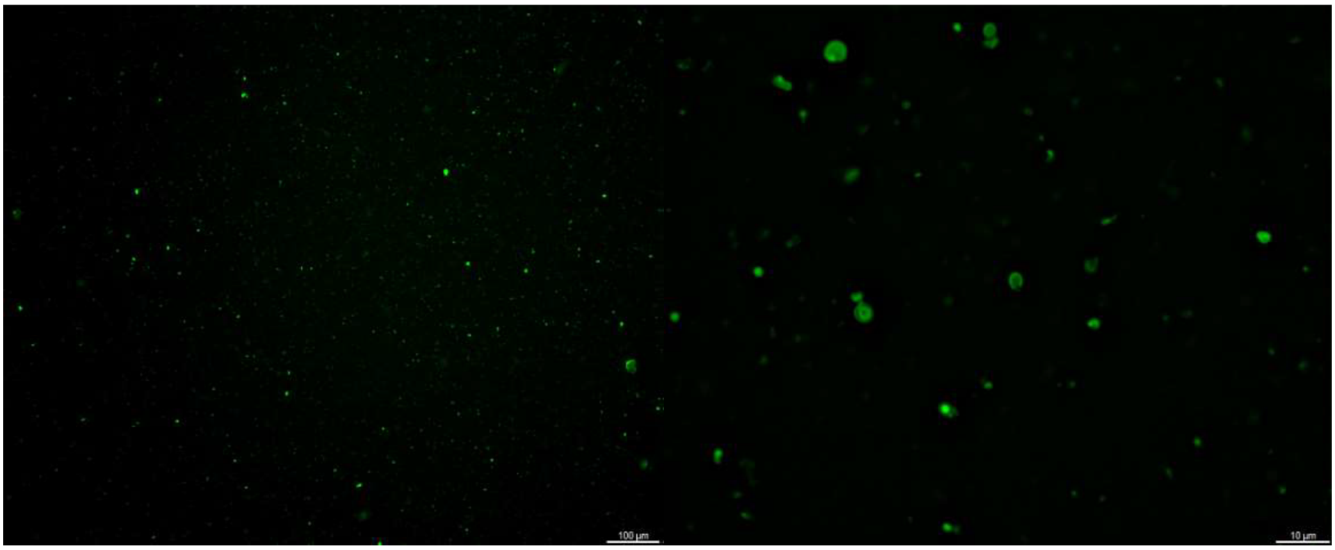

Figure S2. Example of optical microscopy pictures of prepared fiber-hydrated liposomes (fiber-HL1).

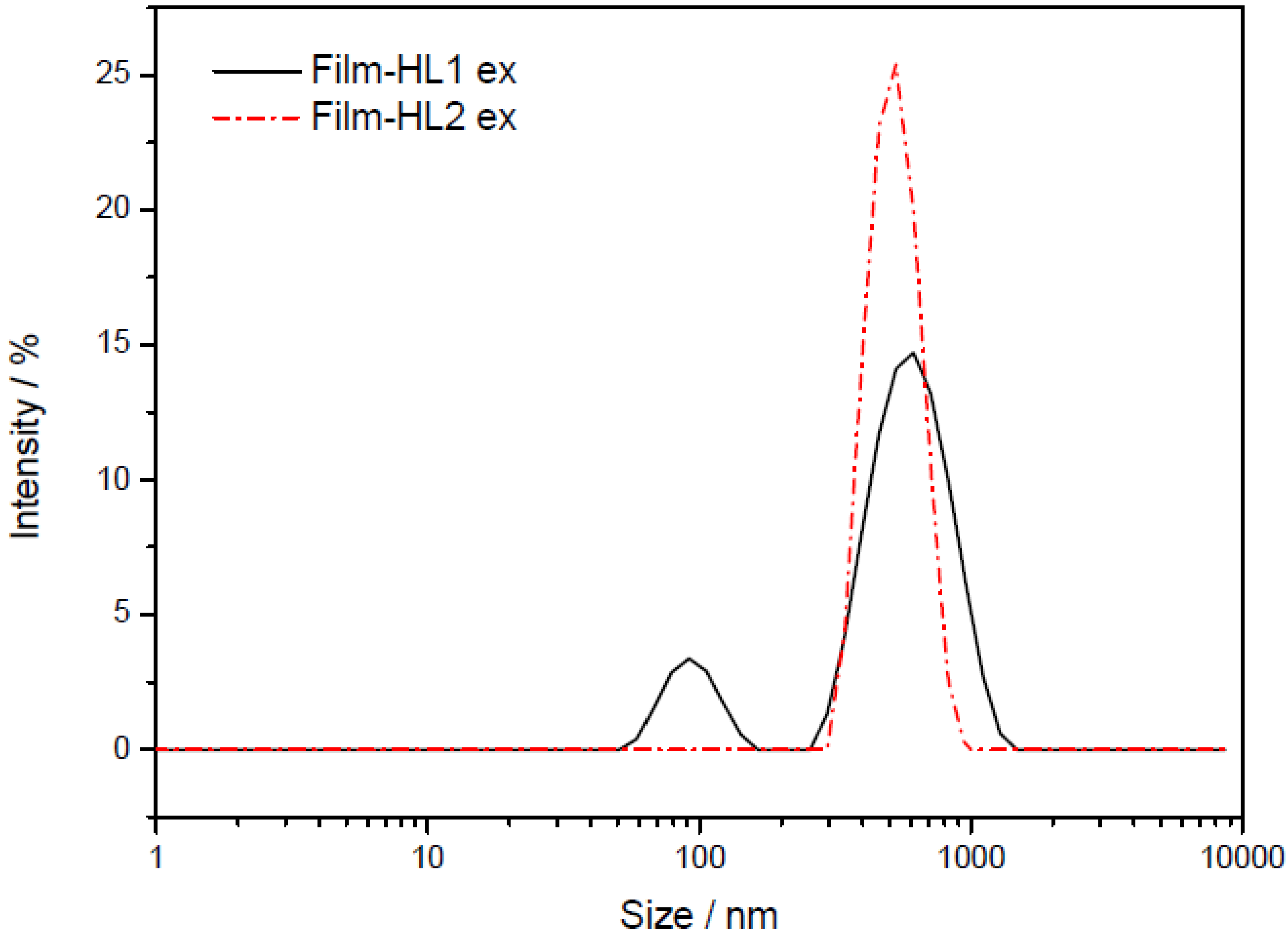

Figure S3. Particle size distribution of film-hydrated liposomes (film-HL) with different chloramphenicol concentrations (CAM). Key: film-HL1- film hydrated liposomes consisting lower amount of CAM (11.1%); film-HL2- film hydrated liposomes consisting higher amount of CAM (62.5%); ex- syringe-extruded liposomes.

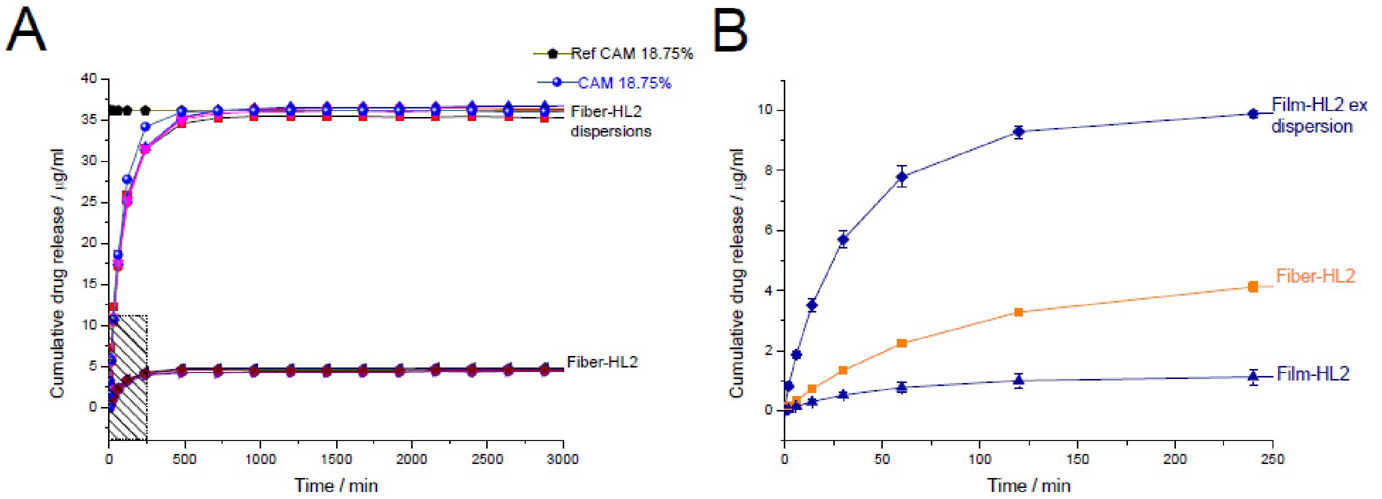

Figure S4. (A) fiber hydrated liposome dispersions with higher CAM concentration (fiber-HL2) and fiber-HL2 samples up to 3000 minutes timepoint; (B) ultracentrifuged and redispersed fiber-HL2 and film-HL2 samples up to 250 minutes timepoint. As references, solutions with 18.75% and 62.5% CAM concentrations were used directly and inserted into the membrane, and their behavior was monitored over time. For clearance purposes the reference CAM solution profiles are not shown in figure B. Key: CAM solution 18.75%- CAM solution (36.2 µg/mL) inserted into the membrane and in a dissolution bath at 37°C; fiber-HL – ultracentrifuged fiber-hydrated liposomes (resuspended in water and inserted into a membrane); film-HL – ultracentrifuged film-hydrated liposomes (resuspended in water and inserted into a membrane); film-HL2ex- filter extruded film-hydrated liposome dispersion with higher CAM concentration inserted into a membrane; Ref fiber-HL2- CAM solution with a theoretical CAM concentration of 18.75% kept in a dissolution bath at 37°C. Graded area shows the region that is enlarged in figure B.

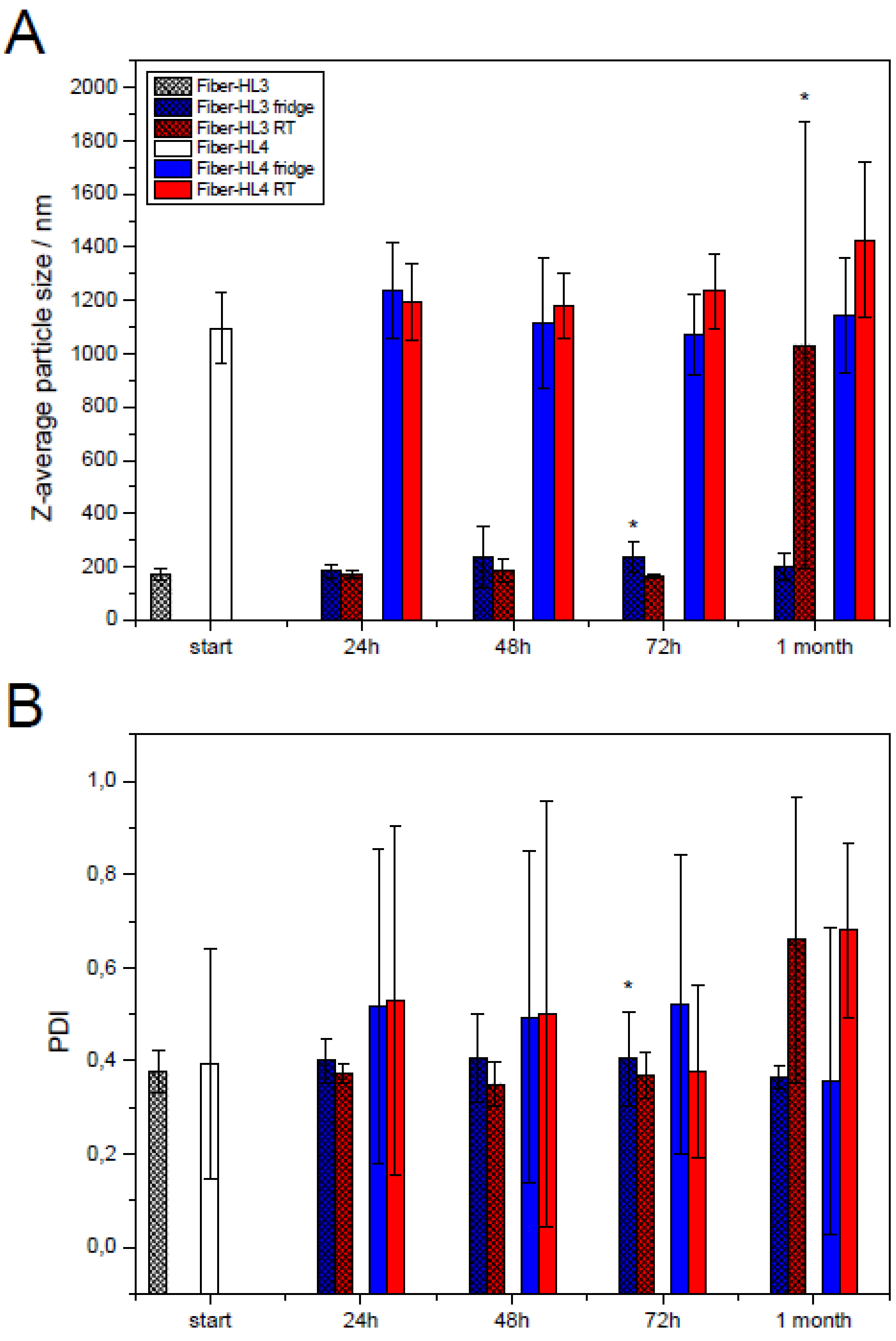

Figure S5. Mean size (A) and PDI (B) of self-formed fiber-hydrated liposomes (fiber-HL3 and fiber-HL4) at different time points when stored at room and fridge temperatures (N=3). Error bars show the standard deviation (SD). Statistically significant differences are shown with asterisk* ($p \leq 0.01$). Key: Fiber-HL- fiber hydrated liposomes, fridge- samples stored in fridge; PDI- polydispersity index; RT- samples stored at room temperature.
